# A Rare Anatomical Variant of Unilateral Piriformis Muscle Agenesis: A Case Report

**DOI:** 10.7759/cureus.4887

**Published:** 2019-06-11

**Authors:** Antonio P Caetano, Leanne L Seeger

**Affiliations:** 1 Radiology, Hospital Center of Lisbon Central, Lisbon, PRT; 2 Radiology, David Geffen School of Medicine at University of California Los Angeles, Los Angeles, USA

**Keywords:** magnetic resonance imaging, piriformis muscle, anatomical variant, sciatic nerve

## Abstract

Anatomical variation of neuromuscular structures of the gluteal region is common. The piriformis muscle, in particular, has an important relationship with the sciatic nerve and may be associated with distinct clinical conditions. We report an incidental finding of unilateral piriformis muscle agenesis diagnosed on computed tomography and magnetic resonance imaging, a rare anatomical variant of the gluteal region.

## Introduction

The piriformis muscle is located in the gluteal region, in close proximity to relevant neuro-vascular structures. It functions as a lateral rotator when the thigh is extended and abductor of the hip when the thigh is flexed [1]. Variations of the piriformis muscle and sciatic nerve anatomy are common. In a meta-analysis published in 2017, Smoll found a prevalence of piriformis and sciatic nerve anomaly of 16.9% in cadavers and 16.2% in surgical cases [2].

## Case presentation

A 57-year-old male with a past medical history of gastroesophageal reflux disease and migraines presented with complaints of left low buttock pain of four weeks duration. The patient stated that the pain progressively worsening, varying from 4 to 10 on the numerical rating scale. The pain radiated down the posterior aspect of the leg to the ankle, with tingling on the dorsum of the foot and toes. The pain was aggravated by activities such as sitting, coughing, standing, sneezing, or laying down. There was no associated weakness and no symptoms of bladder incontinence or dysuria. Physical examination was unremarkable except for point tenderness over the ischiogluteal bursa region on the left.

Pelvic radiographs were performed and revealed no significant abnormalities. The sacroiliac and hip joints were normal.

**Figure 1:**
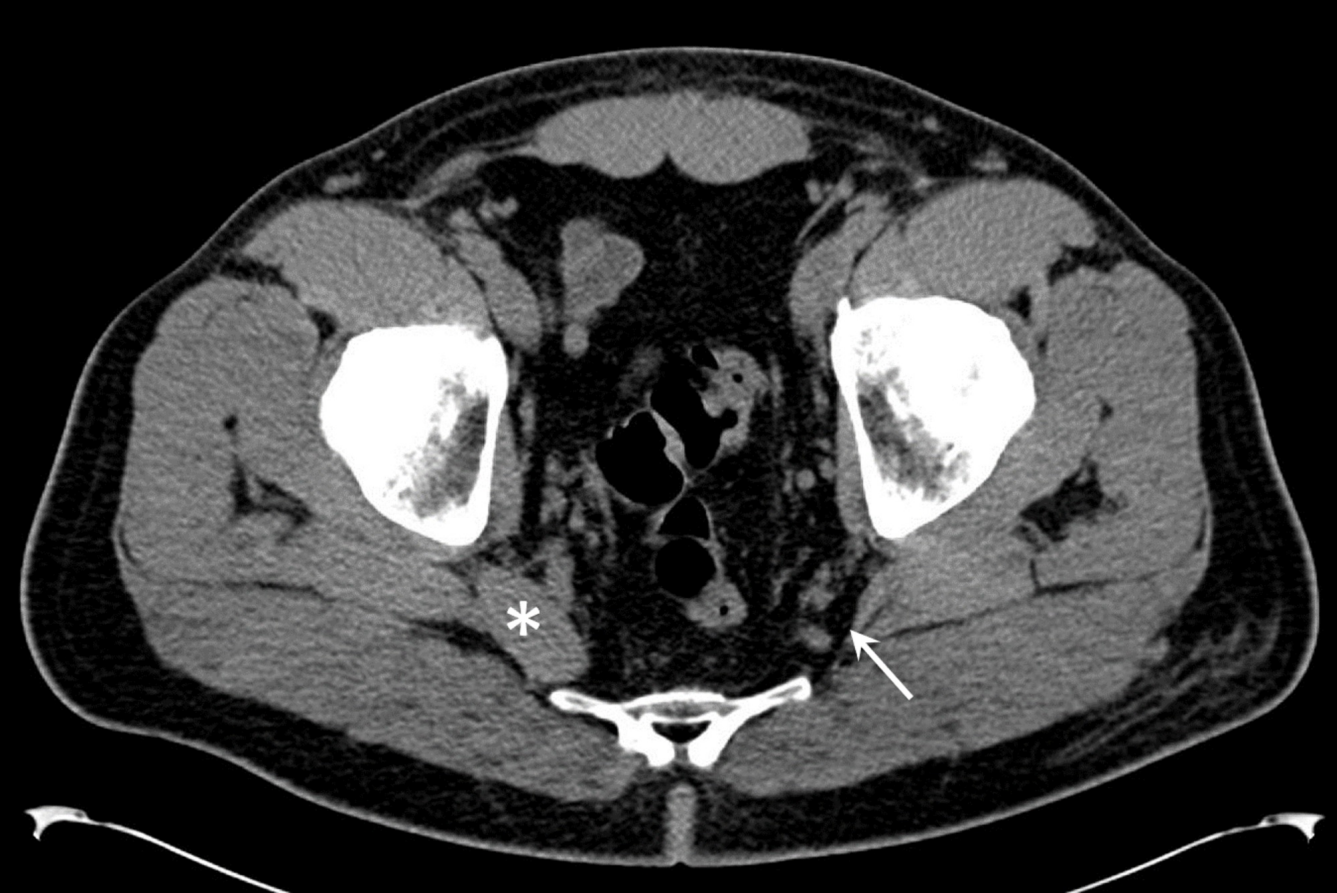
Computed tomography evaluation of the piriformis muscle. Axial computed tomography slice at the pelvic floor level shows absence of piriformis muscle (arrow). The right piriformis muscle is unremarkable (asterisk).

**Figure 2:**
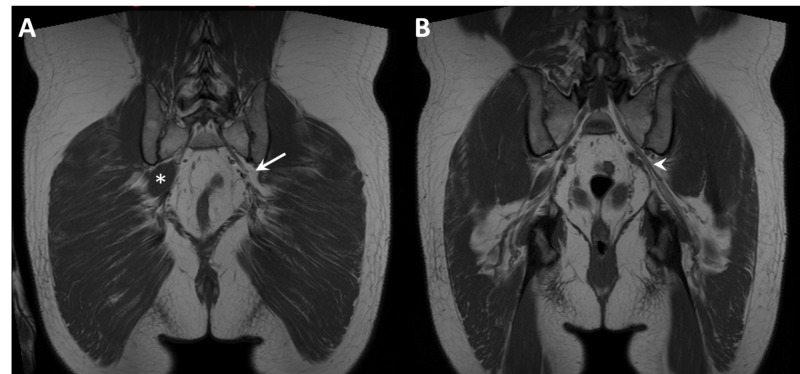
Magnetic resonance evaluation of the piriformis muscle. T1 weighted coronal magnetic resonance image slices show agenesis of the piriformis muscle on the left (A, arrow) and the normal right piriformis muscle (asterisk). The left sciatic nerve is highlighted (B, arrowhead).

Pelvic computed tomography and magnetic resonance imaging were subsequently performed. Neither study revealed a cause for pain, but both incidentally showed complete agenesis of the left piriformis muscle (Figure 1, 2). There were no findings of pathology at or around the deep gluteal space, and the left proximal sciatic nerve was unremarkable.

## Discussion

The piriformis muscle is part of the short rotators of the femur, along with the obturator internus and gemelli [1]. The piriformis muscle originates on the sacrum (anterior or anterolateral aspect of the first or second to fourth sacral segments), gluteal surface of the ilium, and the capsule of the sacroiliac joint. It receives fibers from the sacrotuberous ligament [3-4]. The muscle is often comprised of an upper and lower belly, which fill most of the greater sciatic foramen and is thus in close proximity with branches of the sacral plexus and internal iliac vessels [5]. The gluteal nerves, gluteal vessels, sciatic nerve, and posterior femoral cutaneous nerve typically pass below the piriformis muscle.

The piriformis muscle fascicles run laterally, anteriorly and inferiorly into the gluteal region, posterior to the hip joint and converge to form a rounded tendon. There is a notable discrepancy in the literature regarding the tendon insertion, but according to Windisch et al., it reaches the medial side of the upper border of the greater trochanter at the piriform fossa [1].

The sciatic nerve usually emerges above the piriformis muscle and typically runs below it at the greater sciatic foramen. The relationship of the piriformis muscle and the sciatic nerve has been described by Beaton and Anson, who demonstrated 6 anatomical variants (types A-F), later confirmed by other authors [6-7]. These can be readily identified on MRI [8].

Anatomical variations of the piriformis muscle have been reported in the literature and include a bifid or bipartite muscle, subdivision into several fascicles, tendon or muscle fusion with the gluteus medius, gluteus minimus, superior gemellus, obturator internus and/or joint capsule, as well as a bimuscular conglomeration between the gluteus maximus and piriformis muscle [1-2,9-14]. The exact point of mergence between the two bellies to form the tendon is quite variable [1]. The presence of a double tendon has been reported [15-16]. Also, the distance from the myotendinous junction to the tendon insertion point is variable. An accessory piriformis muscle with a separate tendon has also been described [4,16]. To our knowledge, the absence (agenesis) of the piriformis muscle has only been described previously in a single case report [5]. 

## Conclusions

Piriformis muscle anatomy is variable and has important implications for clinicians and surgeons who deal with the gluteal region. It is necessary to be aware of such variations to safely perform procedures such as imaging-guided injections, total hip arthroplasty, and piriformis tenotomy.
